# Biocompatibility and biodegradation studies of a commercial zinc alloy for temporary mini-implant applications

**DOI:** 10.1038/s41598-017-15873-w

**Published:** 2017-11-15

**Authors:** M. Bobby Kannan, Corey Moore, Shruti Saptarshi, Sahadev Somasundaram, Mohamed Rahuma, Andreas L. Lopata

**Affiliations:** 1 Biomaterials and Engineering Materials (BEM) Laboratory, James Cook University, Townsville, Queensland 4811 Australia; 2 Molecular Allergy Research Laboratory, Department of Molecular & Cell Biology James Cook University, Townsville, Queensland 4811 Australia

## Abstract

In this study, the biocompatibility and *in vitro* degradation behaviour of a commercial zinc-based alloy (Zn-5 Al-4 Mg) were evaluated and compared with that of pure zinc for temporary orthopaedic implant applications. Biocompatibility tests were conducted using human alveolar lung epithelial cells (A549), which showed that the zinc alloy exhibits similar biocompatibility as compared to pure zinc. *In vitro* degradation evaluation was performed using weight loss and electrochemical methods in simulated body fluid (SBF) at 37 °C. Weight loss measurements revealed that the degradation of the zinc alloy was slightly lower during the initial immersion period (1–3 days), but marginally increased after 5 and 7 days immersion as compared to pure zinc. Potentiodynamic polarisation experiments showed that the zinc alloy exhibits higher degradation rate than pure zinc. However, electrochemical impedance spectroscopy analysis suggests that pure zinc is susceptible to localized degradation, whereas the zinc alloy exhibited passivation behaviour. Post-degradation analysis revealed localized degradation in both pure zinc and the zinc alloy.

## Introduction

The emerging interest in biodegradable implants for short-term service life in orthopaedics aims to produce biomaterials with desirable biodegradability, biocompatibility and mechanical properties closer to natural bone. In recent years, a significant amount of research has been undertaken on biodegradable metals, mainly on magnesium-based materials^1^. Magnesium is highly biocompatible, but its undesirably high degradation rate in physiological conditions is a huge disadvantage. Hence, the recent research focus in this field has been on controlling the degradation rate of magnesium by alloying and/or surface coatings^1–6^.

Metallic zinc is a potential biodegradable and biocompatible material for temporary orthopaedic mini-implants such as screw, pins and plates. As an essential nutrient, zinc has many important biological functions, including development and sustenance of bones^7^, food intake and growth^8^, wound healing^9^, cell proliferation and division, and DNA stabilisation and replication^8,10^. Dietary zinc is metabolically absorbed through the small intestine as zinc ions and amnio acid complexes and it is regulated by metallothionein^11^. In the short-term, zinc in the body is regulated to organs such as pancreas, liver, kidneys and spleen^12,13^. However, in the long-term, 90% of the absorbed zinc is deposited in the muscular and skeletal system^14^. The biological half-life of zinc has been determined to be between 162 and 500 days^15,16^, and the daily recommended dose of zinc is 10–15 mg/day^17^. Interestingly, it has been reported that long-term administering of zinc doses ten times the daily recommended intake has produced no adverse effects in humans in relation to wound healing^18^, antirheumatic activity for rheumatoid arthritis^19^ and plasma copper levels^20^. In fact, high concentrations of zinc have been shown to prevent conditions like osteoporosis through promotion of osteoblastogenesis and suppression of osteoclastogenesis^21,22^.

Metallic zinc has physical and mechanical properties similar to those of other common biomaterials: Density = 7.14 g/cm^3^; Young’s Modulus = 70 GPa; Ultimate Tensile Strength (UTS) = 126–246 MPa^23^. The electrochemical dissolution of zinc in aqueous solutions is suggested to occur via the following reactions^24,25^:

1 $$Zn\to Z{n}^{2+}+2{e}^{-}\quad \quad \quad \quad \quad -0.7618\,{{\rm{V}}}_{{\rm{SHE}}}$$

2 $${O}_{2}+2{H}_{2}O+4{e}^{-}\to 4O{H}^{-}\quad \quad +0.4010\,{{\rm{V}}}_{{\rm{SHE}}}$$

where (1) and (2) represent the anodic and cathodic reactions, respectively. However, the degradation mechanism is believed to be largely reliant on even small changes in the electrolyte pH, temperature and composition, and various reaction schemes have been proposed^26,27^. Some principal products of dissolved zinc cations in aqueous solutions are produced via the following reactions:

3 $$Z{n}^{2+}+2O{H}^{-}\to ZnO+{H}_{2}O$$

4 $$Z{n}^{2+}+4O{H}^{-}\to Zn{O}_{2}^{2-}+{H}_{2}O$$

5 $$Z{n}^{2+}+2O{H}^{-}\to Zn{(OH)}_{2}$$

6 $$Z{n}^{2+}+4O{H}^{-}\to Zn{(OH)}_{4}^{2-}$$

Similarly, the selectivity between reactions is governed by the electrolyte conditions. These products are major constituents of the passive films formed on zinc during aqueous corrosion and are known to provide considerable degradation protection since they are thermodynamically stable at room temperature within the pH range 6–12^26^.

As compared to the wealth of literature on the biocompatibility and degradation of magnesium-based material^1–6^, the work done on zinc-based materials is limited. Although extensive research has been done over the past few decades on the corrosion behaviour of zinc and zinc-based alloys (as bulk or coated film) in chloride-containing environments for engineering applications^28–34^, only recently there has been a growing interest on zinc-based materials for potential biodegradable implant applications. Bowen *et al*.^35^ reported that degrading zinc has optimal biocompatibility and the degradation products supress the activities of inflammatory and smooth muscle cells. Liu *et al*.^36^ found that zinc dissolution has no significant destructive effect on erythrocyte. On the other hand, Sherier *et al*.^37^ suggested that free Zn^2+^ ions might hinder cell mobility and adhesion. However, Kubasek *et al*.^38^ reported that the maximum safe Zn^2+^ ion concentrations for U2OS and L929 cell lines are 120 µM and 80 µM, respectively. Bowen *et al*.^39^ examined the *in vivo* degradation behaviour of zinc for absorbable stent applications, and reported the longevity and harmless degradation of zinc metal. They observed that the degradation rate of zinc increases linearly with implantation time. Under short-term *in vivo* condition, zinc oxides were formed, however, after 4.5 to 6 months, calcium phosphate layers were observed. Zinc oxides seem to be inert to the immune system, but depending on the size of these oxide particles can cause cytotoxicity^40^. Drelich *et al*.^41^ reported that defects/cracks in the zinc oxide film increases the degradation rate.

A few binary and ternary zinc alloys (containing magnesium, aluminium, lithium, calcium, copper and/or strontium) have also been studied due to their better mechanical strength as compared to pure zinc. Muni *et al*.^42^ reported that the cell viability (normal human osteoblast cells) for Zn-3Mg was reduced by ~50% at 1 day exposure, but the cells recovered at 3 and 7 days. Dambatta *et al*.^43^ showed that homogenisation of as-cast Zn-3Mg alloy increases the degradation resistance. Vojtĕch *et al*.^44^ reported no significant difference in the degradation rate between pure zinc and zinc alloys (Zn-Mg and Zn-Al-Cu). Interestingly, they found high concentrations of calcium and phosphate in the degradation product layers. Gong *et al*.^45^ and Mostaed *et al*.^46^ reported that extruded Zn-Mg alloys are superior to their cast counterparts in terms of degradation resistance. In contrast, Shen *et al*.^47^ found that the extruded Zn-Mg alloy exhibit lower degradation resistance in comparison with the as-cast alloy. However, Shen *et al*.^47^ and Gong *et al*.^45^ agree that Zn-Mg alloys are biocompatible. Interestingly, aluminium addition to zinc has been reported to cause intergranular degradation^48,49^. On other hand, lithium addition to zinc has improved the degradation resistance and also exhibited excellent biocompatibility^50,51^. Li *et al*.^52^ reported that addition of magnesium, calcium and strontium to zinc can benefit their hemocompatibility and cytocompatibility. However, Liu *et al*.^53^ suggests that calcium or strontium addition to Zn-Mg alloy produced secondary phase particles, which increases galvanic corrosion.

For load-bearing orthopaedic applications, the mechanical integrity of the implant during service is critical. Localized degradation may affect the mechanical integrity of the implant. Unfortunately, zinc undergoes localized degradation in chloride-containing environments^54–56^. Hence, it is important to study the localized degradation susceptibility of zinc in physiological conditions. Literature suggests that the ternary Zn-Al-Mg alloys have superior degradation protection properties in chloride-containing solution than binary system alloys such as Zn-Mg and Zn-Al^57–62^. It should be noted that Zn-Al-Mg alloys are commercially available and have been widely used as galvanizing coating materials on steels due to their high degradation resistance^57,59,61^. This alloy system has other advantages such as better mechanical strength and relatively low density (due to lighter alloying metals such as magnesium and aluminium) as compared to pure zinc for implant applications. Therefore, it is important to understand the biocompatibility and biodegradation behaviour of a Zn-Al-Mg alloy.

In this study, the biocompatibility and biodegradation behaviour of the commercially available Zn-5 Al-4 Mg alloy were examined and compared with that of pure zinc. Weight loss and electrochemical methods were used to evaluate the biodegradation behaviour of the materials in simulated body fluid at 37 °C. Post-degradation analysis was performed using scanning electron microscope (SEM) to identify the mode of degradation.

## Experimental Procedure

The chemical compositions of pure zinc and the commercial Zn-5 Al-4 Mg alloy used in this study are shown in Table 1. The hardness of the materials was measured using a Rockwell hardness tester (Model: Avery Rockwell Hardness Tester, type 6402). For the cytotoxicity testing, human alveolar lung epithelial A549 cells were used. The A549 cells utilised in this study are a human derived epithelial cell line from the lungs and respiratory tract, and is frequently used as indicator of general genotoxicity and cytotoxicity^40^. These cells were obtained from the American Type Culture Collection (ATCC, USA) and maintained in 25 cm^2^ cell culture flasks in an incubator with a humidified atmosphere at 37 °C and 5% CO_2_. The cells were cultured in RPMI-1640 medium (Sigma-Aldrich, USA) supplemented with 10% FBS, 1% penicillin-streptomycin and L-glutamine (Life Technologies, Australia), designated as ‘complete medium’. The cells were cultured to a cell density of 1 × 10^6^ cells/mL before being sub-cultured into fresh media 2–3 times a week. The metal samples were ground with SiC paper up to 2500 grit and later polished with 1 μm alumina powder solution, washed with distilled water and then ultrasonically cleaned in ethanol. Subsequently, the samples were pre-incubated in the complete medium until 96 h at 37 °C in a humidified atmosphere with 5% CO_2_ to obtain the extraction medium, which was used for the cytotoxicity analysis. Metabolic activity of A549 cells exposed to the samples was assessed using the MTS assay which measures the absorbance (490 nm) of the purple dye formazan generated by live cells when exposed to the MTS reagent. (Promega MTS CellTiter 96® aqueous kit, Promega, USA). Briefly, 10,000 cells in 100 µL were seeded into 96-well tissue culture plates (Sarstedt, Germany). After allowing for overnight attachment, the cells were exposed to 100 µL of the extraction medium obtained at 1, 2, 3 and 4-day exposure period. Wells containing cells exposed to the “complete medium” served as positive control. Data were obtained from three independent experiments, each performed in triplicate.

**Table 1:** Composition of pure zinc and zinc alloy (Zn-5 Al-4 Mg), all wt.%.

	Mg	Al	Ca	Fe	Pb	Zn
Pure Zn	<0.001	0.001	<0.001	0.001	0.002	Bal.
Zn Alloy	4.35	4.46	0.035	0.002	0.002	Bal.

In addition, DAPI (4′,6-diamidino-2-phenylindole) staining was carried out to study the changes in nuclear morphology of A549 cells after exposure to the extraction media. A549 cells were allowed to attach overnight on chambered slides (Lab-Tek, Proscitech) at a density of 10^6^ cells per mL and subsequently incubated with the extraction media for up to 4 days. At the end of the incubation period, all cells were collected and washed with Dulbeccos’ phosphate buffered saline (Life Technologies, USA), subjected to fixation and were mounted on Superfrost slides (Proscitech, Australia) using ProLong® Gold Antifade Reagent with DAPI (Molecular Probes, Life Technologies, USA). The slides were subsequently incubated at room temperature for 24 h in the dark before visualization using a Zeiss LSM710 confocal laser scanning microscope (Carl Zeiss, Germany).

*In vitro* degradation behaviour of pure zinc and Zn-5 Al-4 Mg alloy was evaluated by weight loss analysis and electrochemical methods, i.e., potentiodynamic polarisation and electrochemical impedance spectroscopy (EIS), in simulated body fluid (SBF) maintained at a body temperature of 37.5 ± 0.5 °C and pH of 7.4–7.6. The chemical composition of the SBF is given in Table 2 ^63^. Prior to the *in vitro* degradation testing, the samples were ground with SiC paper up to 2500 grit and later polished with 1 μm alumina powder solution, and washed with distilled water and then ultrasonically cleaned in ethanol. In the weight loss testing, the samples were immersed in SBF at a static condition and the weight losses were recorded after 1 to 7 days immersion. Electrochemical experiments were conducted using a potentiostat/galvanostat and a frequency response analyser (Model: ACM Gill AC, ACM Instruments). A typical three-electrode system consisting of graphite as a counter electrode, Ag/AgCl electrode as a reference electrode and the sample as a working electrode was used in this study. The potentiodynamic polarisation experiments were conducted at a scan rate of 0.5 mV/sec. The EIS experiments were performed over the frequency range of 1 × 10^5^ Hz to 1 × 10^−2^ Hz and at an AC amplitude of 5 mV. The EIS data were analysed using equivalent circuit modelling (Software: ZSimpWin v3.21, Princeton Applied Research). All the *in vitro* degradation tests were conducted in triplicate. Scanning electron microscope (SEM) was used to analyse the post-degradation samples.

**Table 2:** Chemical composition of the simulated body fluid (SBF).

Chemical	Amount (/L)
NaCl	8.036 (g)
NaHCO_3_	0.352 (g)
KCl	0.225 (g)
K_2_HPO_4_∙3H_2_O	0.23 (g)
MgCl_2_∙6H_2_O	0.311 (g)
1 M HCl	40 (mL)
CaCl_2_	0.293 (g)
Na_2_SO_4_	0.072 (g)
TRIS buffer^a^	6.063 (g)

^a^TRIS buffer = tris(hydroxylmethylaminomethane).

## Results and Discussion

### Biocompatibility

**Figure 1:**
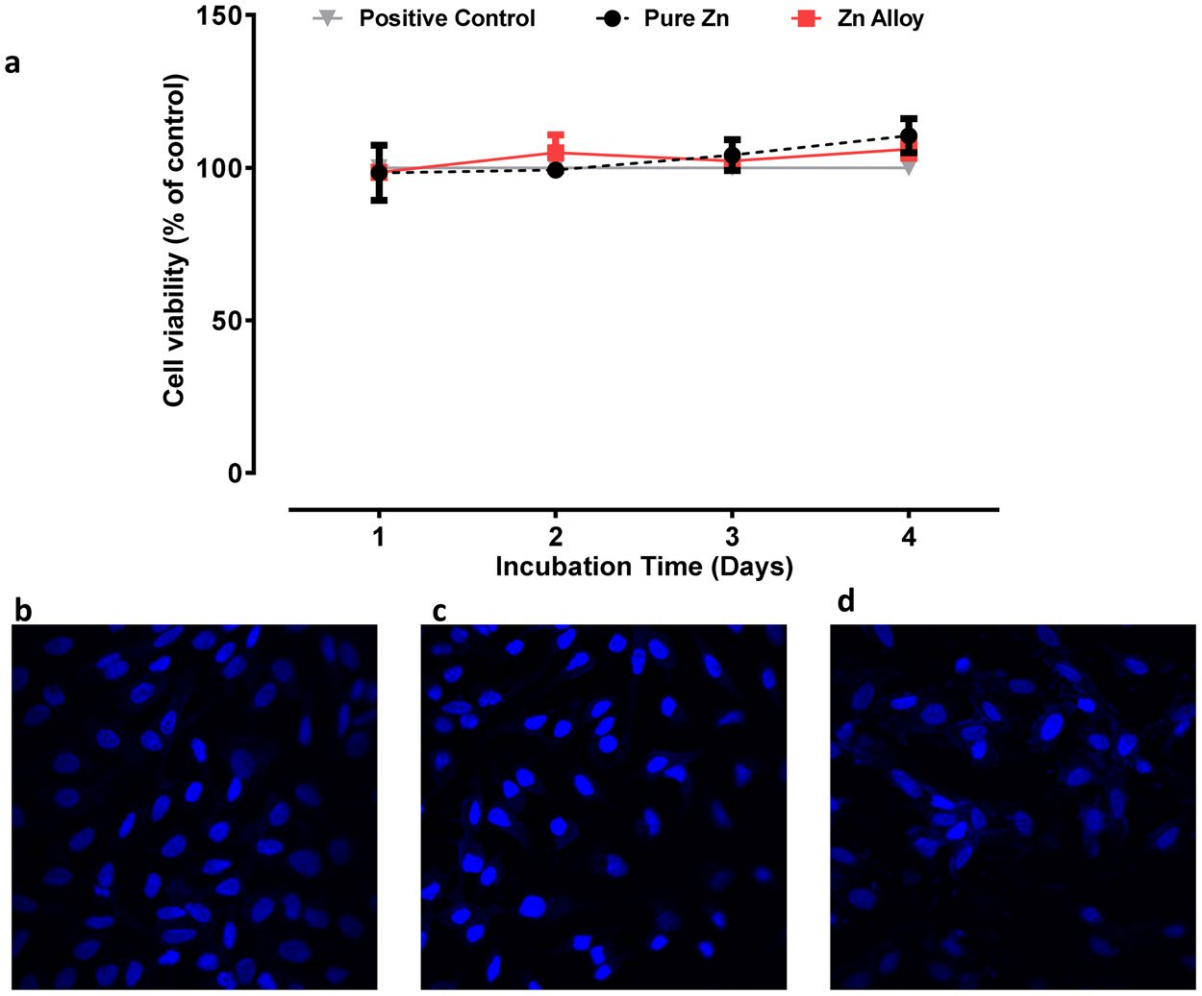
(**a**) Cell viability of pure zinc and zinc alloy exposed to A549 human lung alveolar epithelial cells. Cytotoxic response of human lung epithelial cells exposed to extraction medium was assessed using the MTS assay. Specific absorbance from untreated cells was used as the reference for normalising the test-well data to calculate “% of control cell viability. Values are expressed as mean ±SEM (n = 3 separate experiments each performed in triplicate). CLSM images of DAPI staining of (**b**) cells exposed to complete medium, (**c**) zinc alloy and (**d**) pure zinc, after 4 days exposure to A549 human lung alveolar epithelial cells.

The cell viability (cytotoxicity) of pure zinc and the zinc alloy on A549 cells is shown in (Fig. 1) as compared to cells exposed to the complete medium. A549 cells exposed to the extraction media obtained from the zinc or zinc alloy samples did not demonstrate cytotoxicity at the end of the 4 days testing period. Figure 1b–d show the nuclear morphology of the treated cells after DAPI staining. Normally, cells undergoing apoptosis exhibit characteristic condensation of the nuclear material. In the present study, cells exposed to zinc or the zinc alloy demonstrated nuclear morphology similar to the control cells (exposed to cell culture medium alone) even after the 96 h exposure period, further confirming the non-toxic nature of the samples. These results serve as a preliminary indication of the biocompatibility of pure zinc and the zinc alloy.

### Potentiodynamic polarisation

**Figure 2:**
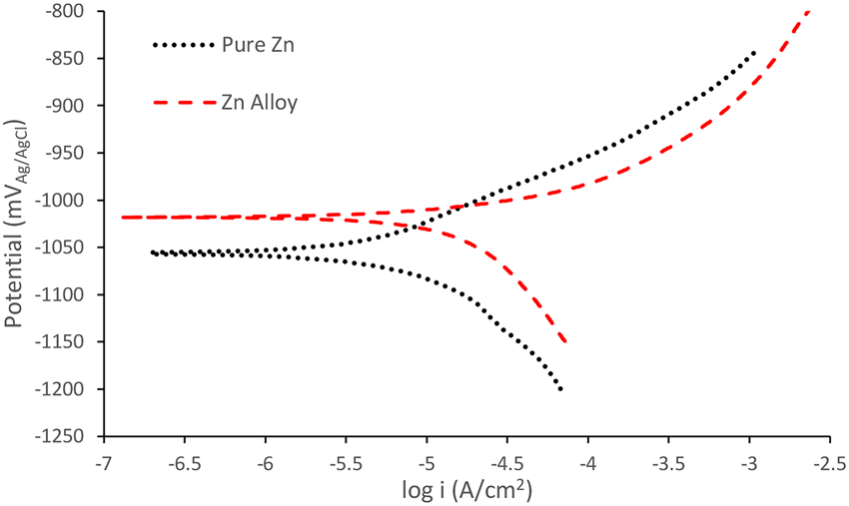
Potentiodynamic polarisation curves of pure zinc and zinc alloy in SBF.

**Figure 3:**
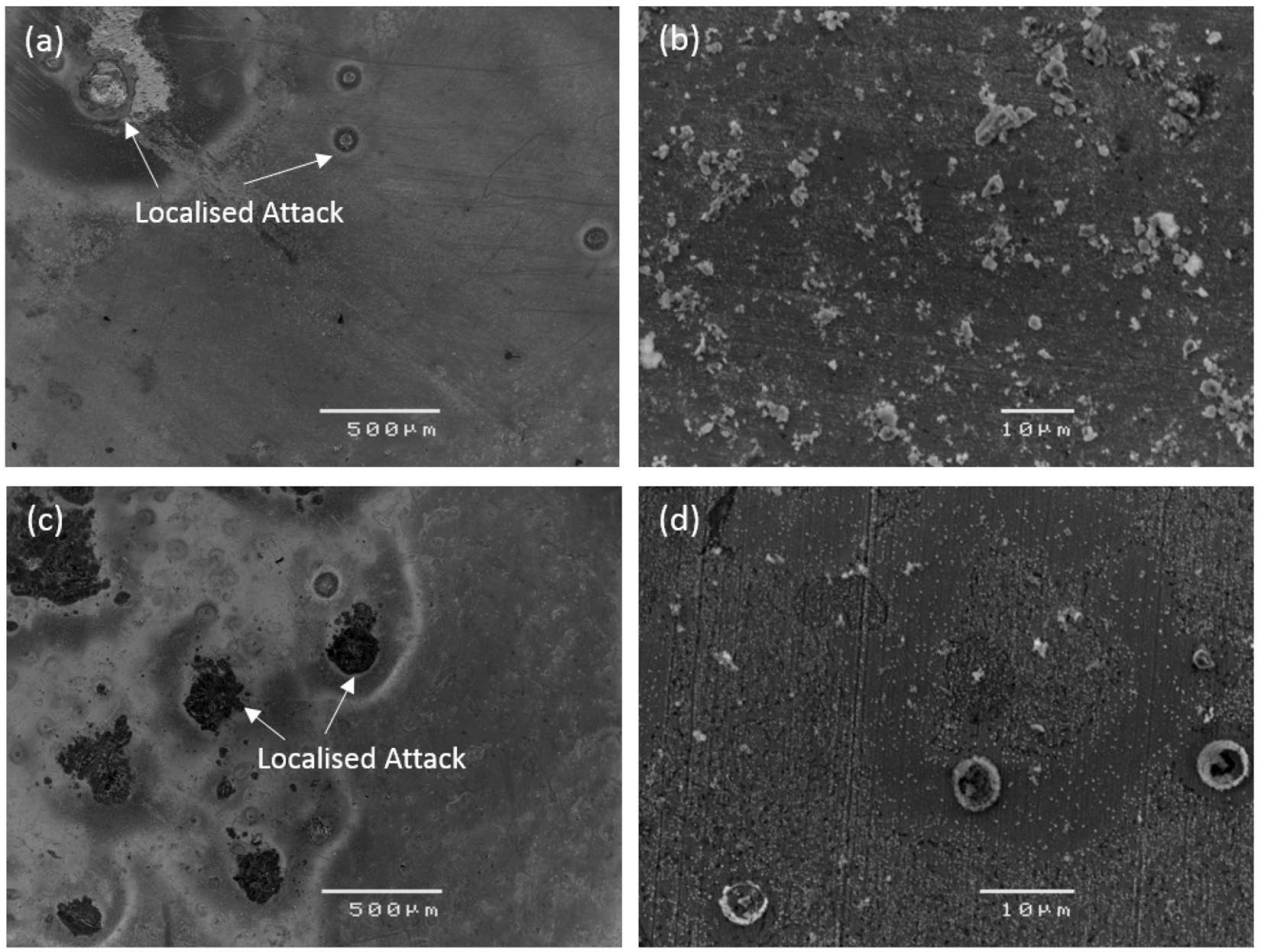
SEM micrographs of: (**a**) & (**b**) pure zinc and (**c**) & (**d**) zinc alloy, after potentiodynamic polarisation in SBF.

The potentiodynamic polarisation curves of pure zinc and the zinc alloy are shown in Fig. 2, and the electrochemical data obtained from the curves are presented in Table 3. The corrosion potential (E_corr_) of the zinc alloy was slightly (~10 mV) more noble as compared to pure zinc. The cathodic polarisation curves suggest that the cathodic activity was higher for the zinc alloy in comparison with pure zinc. This difference in cathodic activity can be attributed to the alloying elements. In the case of the anodic side of the polarisation curves, the dissolution behaviour of the zinc alloy was higher than pure zinc. However, both pure zinc and the zinc alloy did not show any active passive region or breakdown potential. The corrosion current density (i_corr_) calculated from the cathodic curves suggested that the i_corr_ value of the zinc alloy is ~85% higher than pure zinc i.e., 17.7 µA/cm^2^ and 9.55 µA/cm^2^, respectively. The calculated degradation rate for the zinc alloy was 0.32 mm/y and for pure zinc 0.14 mm/y. As expected, the degradation rate of pure zinc and the zinc alloy was significantly lower than that of pure magnesium (degradation rate = 0.54 mm/y; i_corr_ = 23.5 µA/cm^2^)^64^. Post-polarisation SEM micrographs of pure zinc and the zinc alloy are shown in Fig. 3. The morphology of pure zinc revealed localized attack (Fig. 3a and b). In the case of the zinc alloy, the localized attack increased, as demonstrated by the relative larger areas of evident damage (Fig. 3c and d).

**Table 3:** Electrochemical data obtained from the potentiodynamic polarisation curves of pure zinc and zinc alloy (Zn-5 Al-4 Mg).

	E_corr_ (mV_Ag/AgCl_)	β_a_ (mV/decade)	β_c_ (mV/decade)	i_corr_ (μA/cm^2^)	Corrosion Rate (mm/y)
Pure Zn	−1032 ± 5	318	−417	9.55 ± 1.1	0.14
Zn Alloy	−1020 ± 5	295	−202	17.7 ± 1.2	0.32

Values represent means of triplicate samples ± absolute standard deviations.

### EIS

**Figure 4:**
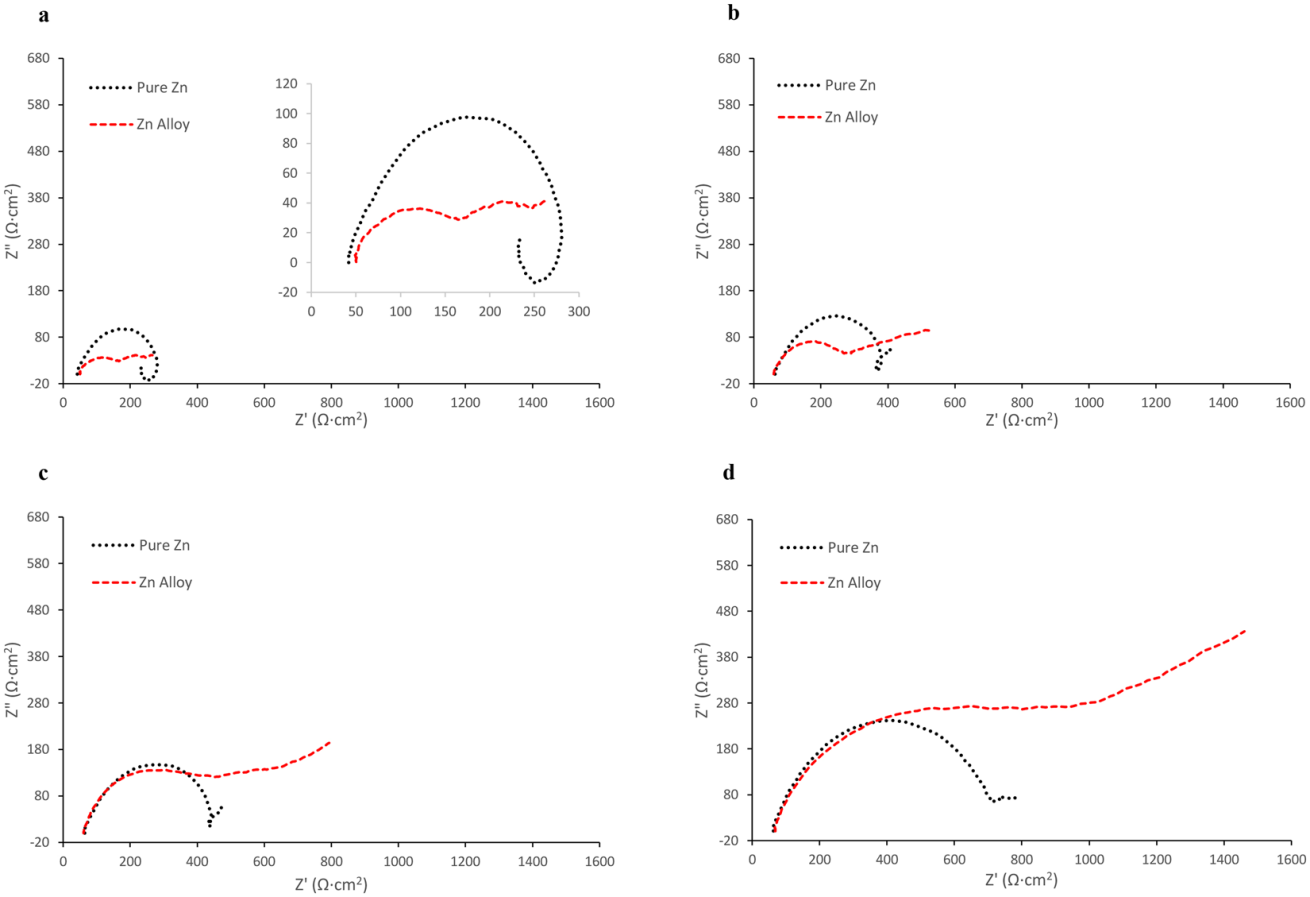
Nyquist plots of pure zinc and zinc alloy after: (**a**) 2 hours, (**b**) 1 day, (**c**) 2 days and (**d**) 3 days immersion in SBF.

**Figure 5:**
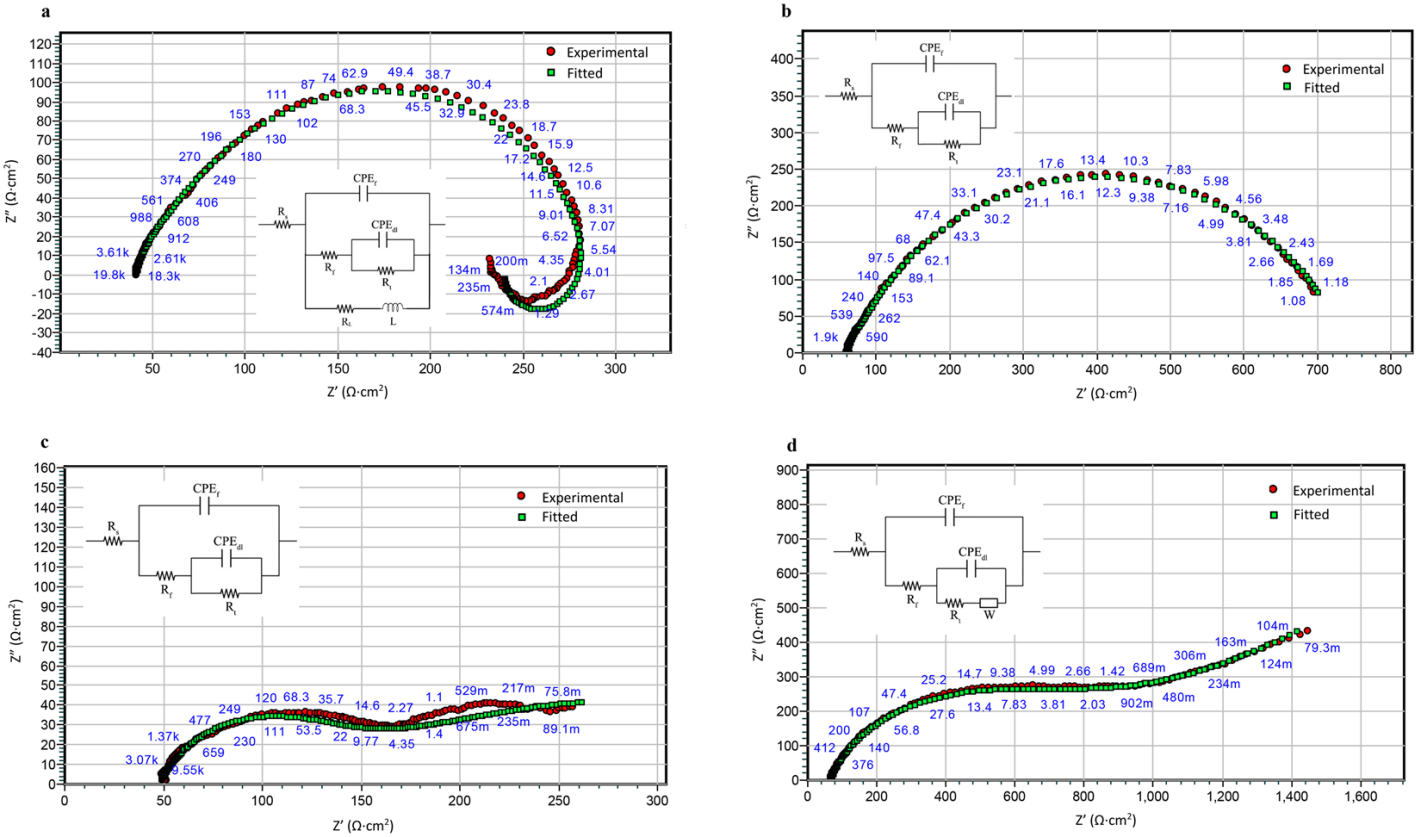
EIS fitting of experimental data for: (**a**) pure zinc after 2 hours, (**b**) pure zinc after 72 hours, (**c**) zinc alloy after 2 hours and (**d**) zinc alloy after 72 hours immersion in SBF. (Insets: Equivalent circuits).

The EIS spectra for pure zinc and the zinc alloy over 72 h immersion in SBF is shown in Fig. 4. The equivalent circuits (EC) models used and the fitting for pure zinc and the zinc alloy after 2 h and 72 h immersion are shown in Fig. 5. The data obtained from EIS modelling are presented in Table 4. After 2 h immersion, pure zinc showed a capacitive loop and an inductive loop. The low frequency inductive loop is a general indication of localized degradation^65,66^ or adsorption of intermediate corrosion products or ions onto the surface^67,68^. The zinc alloy showed two capacitive loops, but no inductive loop. The high frequency capacitive loop can be attributed to charge transfer resistance and the mid-frequency capacitive loop is related to the film resistance. The EC model used for zinc alloy consisted of the following elements: R_s_ (solution resistance), R_ct_ (charge transfer resistance), CPE_dl_ (double layer capacitance) and R_f_ (film resistance). For the pure zinc, which exhibited an inductive loop, L (inductance) and CPE_f_ (capacitance due to film effect) elements were added. The polarisation resistance (R_p_) of the samples was calculated by adding the R_ct_ and R_f_. The R_p_ of the zinc alloy after 2 h exposure to SBF was 63% higher than that of pure zinc (pure zinc = 250.56 Ω∙cm^2^; zinc alloy = 408.39 Ω∙cm^2^).

**Table 4:** EIS spectra equivalent circuit modelling data of pure zinc and zinc alloy (Zn-5 Al-4 Mg).

	Immersion Time	R_f_ (Ω∙cm^2^) (x 10^2^)	CPE_f_ (Ω^−1^∙cm^−2^∙s^−n^) (x 10^−5^)	n	R_t_ (Ω∙cm^2^) (x 10^2^)	CPE_dl_ (Ω^−1^∙cm^−2^∙s^−n^) (x 10^−5^)	n	R_L_ (Ω∙cm^2^) (x 10^2^)	L (Ω∙cm^2^∙s)	W (Ω^−1^∙cm^−2^∙s^−0.5^) (x 10^2^)	R_p_ (Ω∙cm^2^) (x 10^2^)
Pure Zn	**2 h**	0.88 ± 0.09	1.40 ± 0.18	0.87	1.62 ± 0.1	2.09 ± 0.14	0.85	9.58 ± 0.47	109 ± 7.317	—	**2.5** ± 0.17
	**1d**	0.91 ± 0.06	0.79 ± 0.29	0.8	2.27 ± 0.35	4.29 ± 0.32	0.86	—	—	—	**3.19** ± 0.43
	**2d**	0.97 ± 0.02	0.75 ± 0.2	0.99	2.9 ± 0.2	4.73 ± 0.87	0.81	—	—	—	**3.87** ± 0.29
	**3d**	0.96. ± 0.01	0.71 ± 0.1	1	5.93 ± 0.46	6.65 ± 1.06	0.69	—	—	—	**6.9** ± 0.5
Zn Alloy	**2 h**	0.83 ± 0.06.45	5.86 ± 2.11	0.74	3.25 ± 0.55	607.12 ± 69.61	0.32	—	—	—	**4.08** ± 0.5
	**1d**	2.26 ± 0.76	13.27 ± 1.07	0.69	3.36 ± 0.42	899.67 ± 234.09	0.63	—	—	—	**5.63** ± 1.26
	**2d**	2.89 ± 1	8.11 ± 1.09	0.79	11.68 ± 0.78	283.10 ± 38.15	0.36	—	—	3.14 ± 0.49	**14.57** ± 1.75
	**3d**	2.17 ± 0.3	51.26 ± 17.33	0.18	26.82 ± 1.83	21.41 ± 3.61	0.79	—	—	0.08 ± 0.01	**28.99** ± 2.04

Values represent means of triplicate samples ± absolute standard deviation.

After 24 h exposure, the R_p_ of the zinc alloy increased by 38%. In the case of pure zinc, the inductive loop disappeared and the R_p_ increased by 27% (R_p_ = 319.61 Ω∙cm^2^). However, after 48 h exposure, the zinc alloy continued to display passivation effect, but the mid-frequency capacitive loop has transformed to Warburg impedance. This type of behaviour has been reported in the literature for zinc metal^69^. The significance of a Warburg impedance is the presence of a porous passive film facilitating diffusion controlled processes^69,70^. A Warburg diffusion element was used to model the EIS spectra for the zinc alloy. The R_p_ of the zinc alloy was ~4 times higher than pure zinc (pure zinc = 387.36 Ω∙cm^2^, zinc alloy = 1457.2 Ω∙cm^2^) after 48 h exposure. The trend continued even after 72 h exposure, the R_p_ values for pure zinc and zinc alloy were 690.13 Ω∙cm^2^ and 2899.66 Ω∙cm^2^, respectively.

### Weight Loss

**Figure 6:**
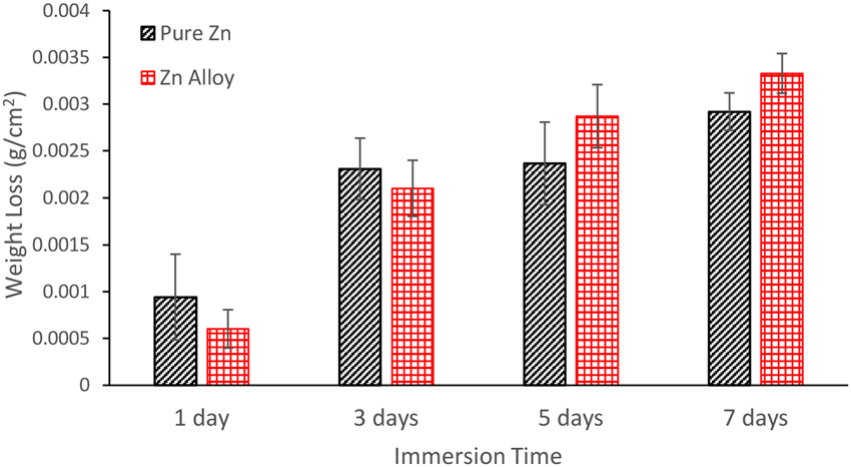
Weight loss results for pure zinc and zinc alloy over 7 days immersion in SBF. (Each bar represents the mean of a triplicate sample, and error bars represent standard deviations).

**Figure 7:**
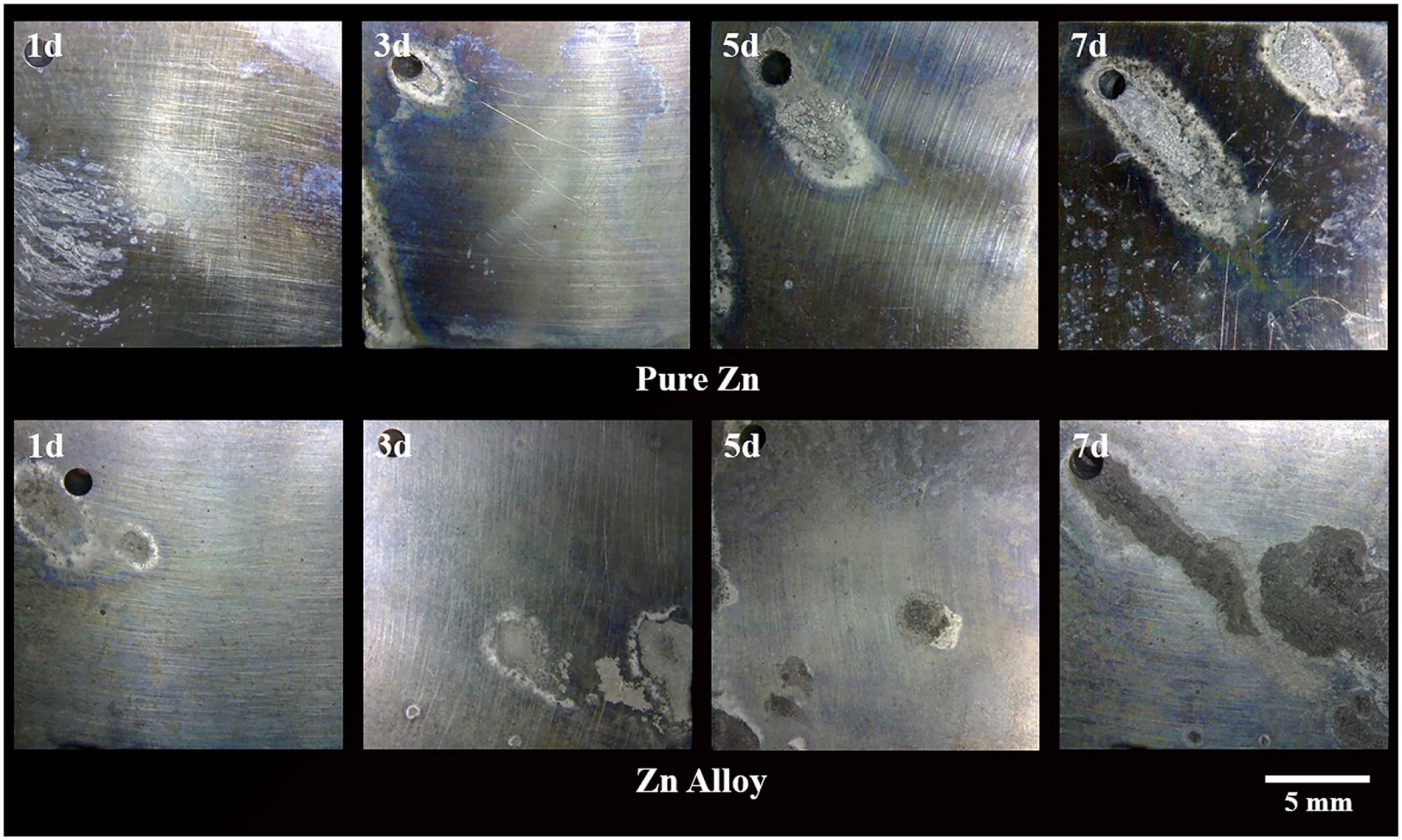
Macrographs of pure zinc and zinc alloy after 1, 3, 5 and 7-day immersion in SBF.

The weight loss measurements for pure zinc and the zinc alloy are shown in Fig. 6. As expected, the weight loss increased with increasing exposure. Interestingly, the weight loss data for the zinc alloy were not remarkably different to the pure zinc during 1 to 7 days immersion in SBF. After 1 and 3 days immersion, the weight loss of the alloy was marginally lower than pure zinc, but after 5 and 7 days the trend reversed. The macrographs of pure zinc and the zinc alloy after each interval of immersion are shown in Fig. 7. Both pure zinc and the zinc alloy have undergone localized degradation and the intensity has increased with increasing immersion time. It was interesting to note that the pitting nucleation was not high, but the growth of pits was very rapid. After 7 days immersion, the localized degradation attack was remarkably high in both pure zinc and the zinc alloy. The overall degradation rates derived from the weight loss test were 0.31 mm/y for pure zinc and 0.35 mm/y for the zinc alloy.

### Mechanism

The EIS spectra suggests that the zinc alloy exhibits passivation behaviour. This can be attributed to the alloying elements in the zinc alloy, especially aluminium. Literature on the corrosion behaviour of aluminium-containing zinc alloy coatings, e.g., Zn-Al^71^ and Zn-Al-Mg^61,72^, in chloride-containing solution suggests that aluminium forms a thick and complex layers. Volovitch, *et al*.^62^ reported that aluminium formed basic aluminium-oxides in the initial stages of corrosion of a Zn-Al-Mg alloy. Studies have also suggested that aluminium has a lower dissolution tendency as compared to zinc and magnesium in Zn-Al-Mg alloy system^60,62^. On the other hand, magnesium forms magnesium hydroxide in aqueous solutions, which is a protective film, but in chloride-containing solution, the protective film converts to soluble magnesium chloride, as shown below^73^.

7 $$Mg(s)+2{H}_{2}O\to Mg{(OH)}_{2}+{H}_{2}$$

8 $$Mg{(OH)}_{2}+2C{l}^{-}\,\to MgC{l}_{2}+2O{H}^{-}$$

In the current study, the Warburg impedance observed under long-term EIS suggests that the film formed on the alloy is porous in nature and introduces diffusion characteristics; hence, the stability of the film in physiological condition was only temporary.

**Figure 8:**
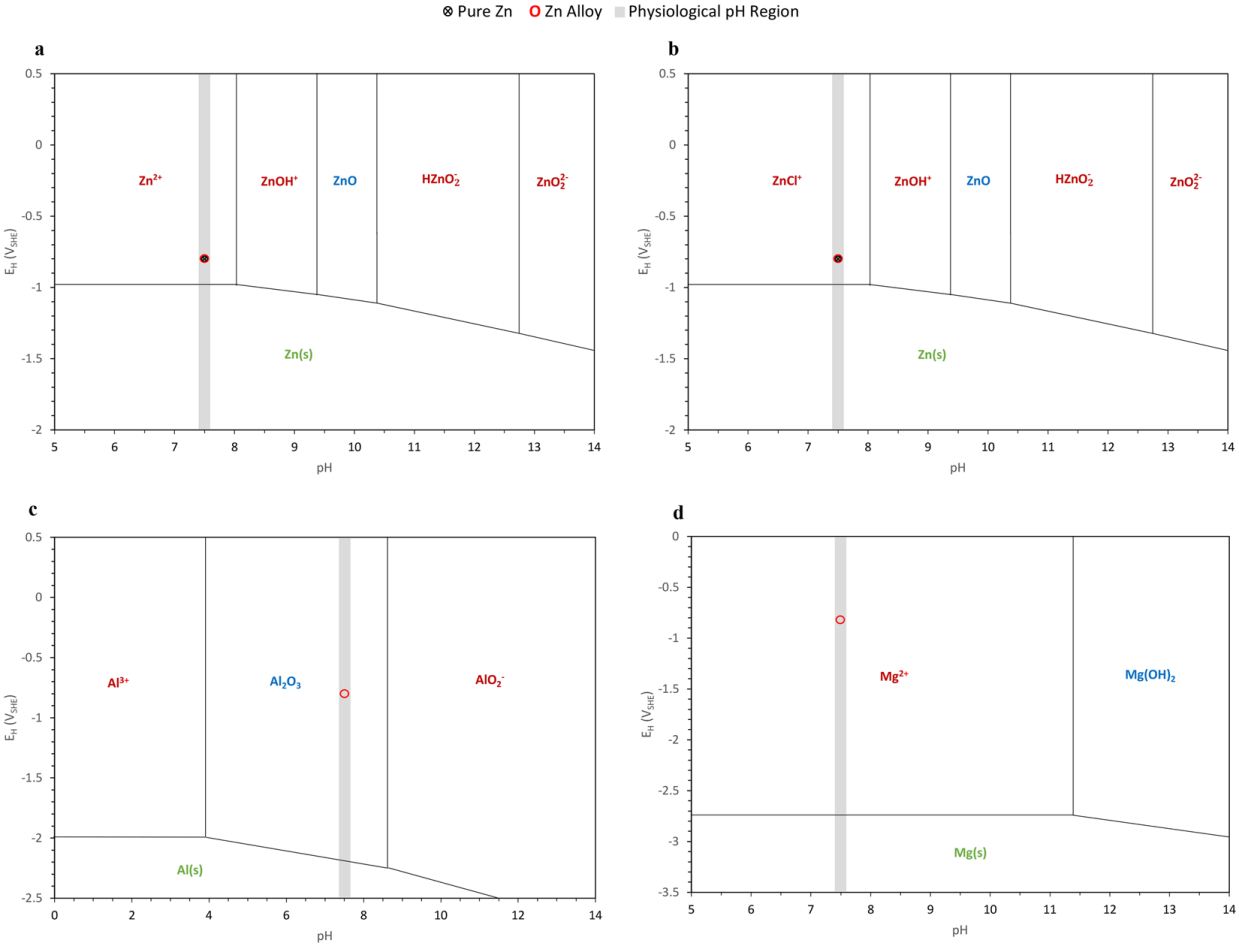
Pourbaix diagram for Zn-C-Cl-H_2_O system at 25 °C with E_corr_ (E_H_) values for pure zinc and zinc alloy and the physiological pH range overlayed (adapted from [74]).

To further understand the passivation behaviour of pure zinc and the zinc alloy in the physiological environment that contains chloride ions, the Pourbaix diagrams of zinc, aluminium and magnesium were used^74^. Figure 8 (a–d) shows the E_corr_ values of pure zinc and the zinc alloy embedded on the Pourbaix diagrams. Although (Fig. 8a) suggests that the potentials of pure zinc and the zinc alloy are in the passive region, the presence of chloride shifts the passivity region towards the higher pH regions and hence undergo dissolution. It can be noted that the experimental conditions confined pure zinc and the zinc alloy to the active ZnCl^+^ region throughout the immersion period (Fig. 8b). Hence, zinc did not show any strong passivation. The passivation observed in the EIS experiments (Fig. 4) of the zinc alloy could be attributed to aluminium, which is stable in the physiological pH range (Fig. 8c). Magnesium is however not stable in that pH range (Fig. 8d).

The potentiodynamic polarisation curves suggest that the cathodic activity of the zinc was higher as compared to pure zinc (Fig. 2). This can be attributed to the alloying elements in the zinc alloy. Although oxygen reduction reaction is the predominant cathodic reaction for zinc metal, hydrogen evolution reaction is feasible in the zinc alloy due to the presence of the alloying elements such as magnesium and aluminium. It should be noted that the hydrogen-evolution exchange current densities of the alloying elements magnesium and aluminium are higher than that of zinc (~10^−8^ −10^−9^, ~10^−10^ and ~10^−11^ A/cm^2^ respectively^75,76^). Therefore, the cathodic current of the alloy was higher than that of pure zinc. The anodic reaction of the zinc alloy was also higher than pure zinc, which could be due to selective leaching of elements under accelerated conditions. Magnesium being more reactive than aluminium and zinc, selective leaching of magnesium could have caused the increase in anodic current during polarisation. However, the EIS experiments, which is non-destructive, revealed passivation behaviour in the alloy. The weight loss method also showed improved degradation resistance of the zinc alloy as compared to pure zinc during the initial immersion period, but exhibited localized degradation with increasing exposure, probably due to galvanic effect.

The study suggests that the zinc alloy exhibited similar biocompatibility to pure zinc. It was interesting to see that the biodegradation resistance of the zinc alloy, which has been used for galvanization for its excellent corrosion resistance, was not superior to that of pure zinc in physiological conditions. During the initial immersion period, the zinc alloy exhibited passivation behaviour, but the passivity became less stable with exposure time and ultimately gave rise to localized degradation similar to pure zinc. However, this zinc alloy has some attractive properties (density and hardness) as compared to pure zinc, which are essential for load-bearing implant applications. Due to the presence of light metals, the density of the zinc alloy is approximately 17% lower than pure zinc (pure zinc = 7.14 g/cm^3^ and zinc alloy = 5.908 g/cm^3^). The hardness of the zinc alloy was approximately 14% higher than pure zinc (pure zinc = 79.2 HRB and zinc alloy = 89.9 HRB). The biocompatibility and the attractive physical and mechanical properties make the commercial zinc alloy a potential material for temporary mini-implant applications. However, surface engineering is essential to delay the localized degradation of the commercial zinc alloy.

## Conclusions

The biocompatibility and *in vitro* degradation behaviour of a commercial zinc alloy (Zn-5 Al-4 Mg) were evaluated and compared with that of pure zinc. The zinc alloy showed similar biocompatibility to pure zinc in the cytotoxicity assay conducted using human alveolar lung epithelial cells (A549). The aluminium content in the alloy improved the passivation behaviour, but was only temporary in the physiological conditions. The potentiodynamic polarisation results suggested that the zinc alloy degradation rate is marginally higher than pure zinc owing to the higher hydrogen exchange current density of the alloying elements (magnesium and aluminium) as compared to zinc. The localized degradation susceptibility of the zinc alloy was similar to pure zinc. In addition to the comparable biocompatibility and biodegradability of the zinc alloy as compared to pure zinc, the alloy exhibits lower density and higher hardness, which make it more attractive for load-bearing orthopaedic applications.
